# Supplementary material for: A galactic microquasar mimicking winged radio galaxies
Source: Nat Commun. 2017 Nov 24;8:1757. doi: 10.1038/s41467-017-01976-5 (PMC5700963; doi:10.1038/s41467-017-01976-5)
Supplement: Supplementary file 1 — Supplementary Information [file 41467_2017_1976_MOESM1_ESM.pdf]

Supplementary Information

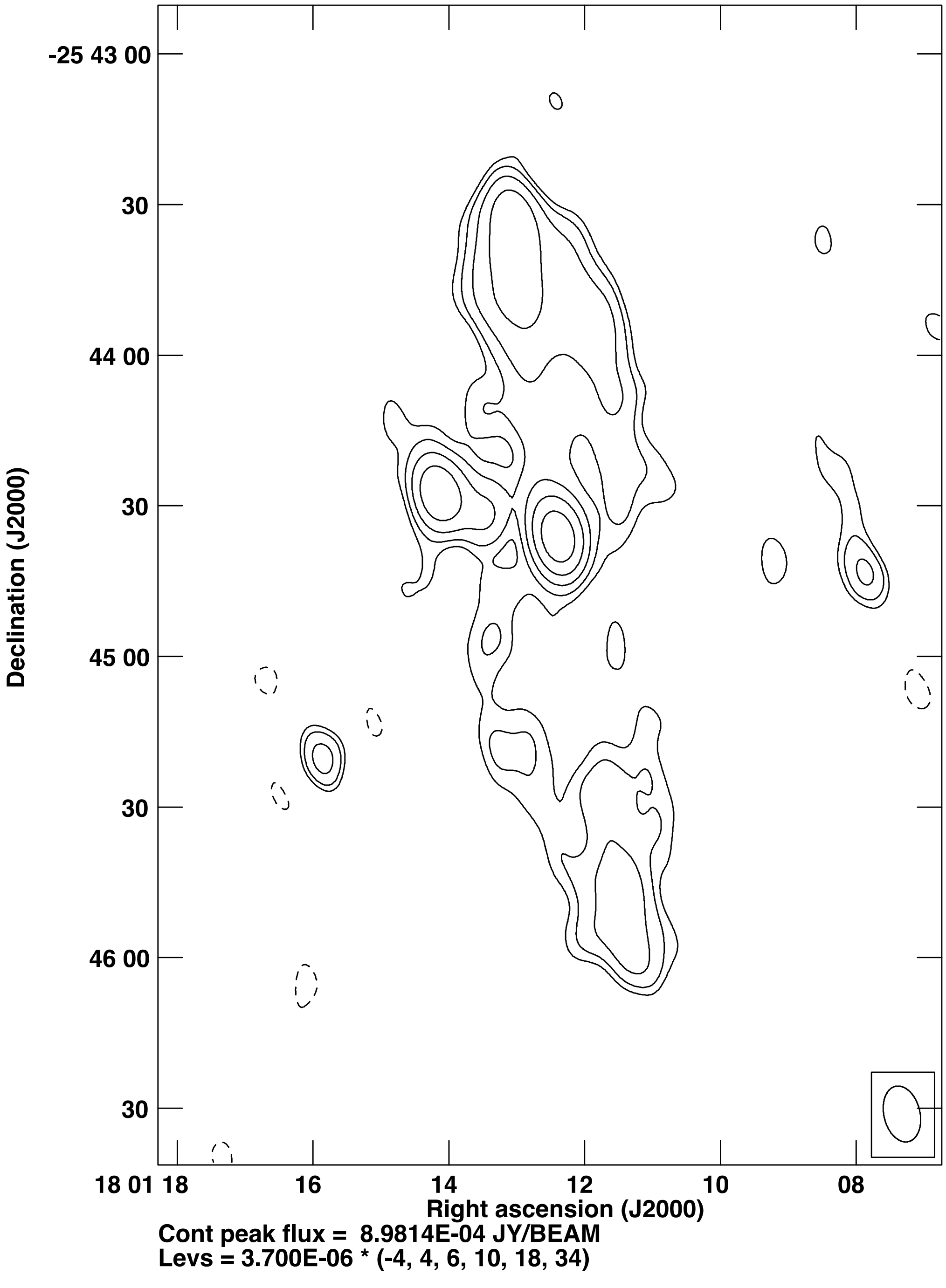

Supplementary Figure 1. Contour map version of Figure 1. The contour levels shown are in rms noise units of 3.7 μJy beam^{-1}. Their increment follows a geometric progression starting at the 4σ level.

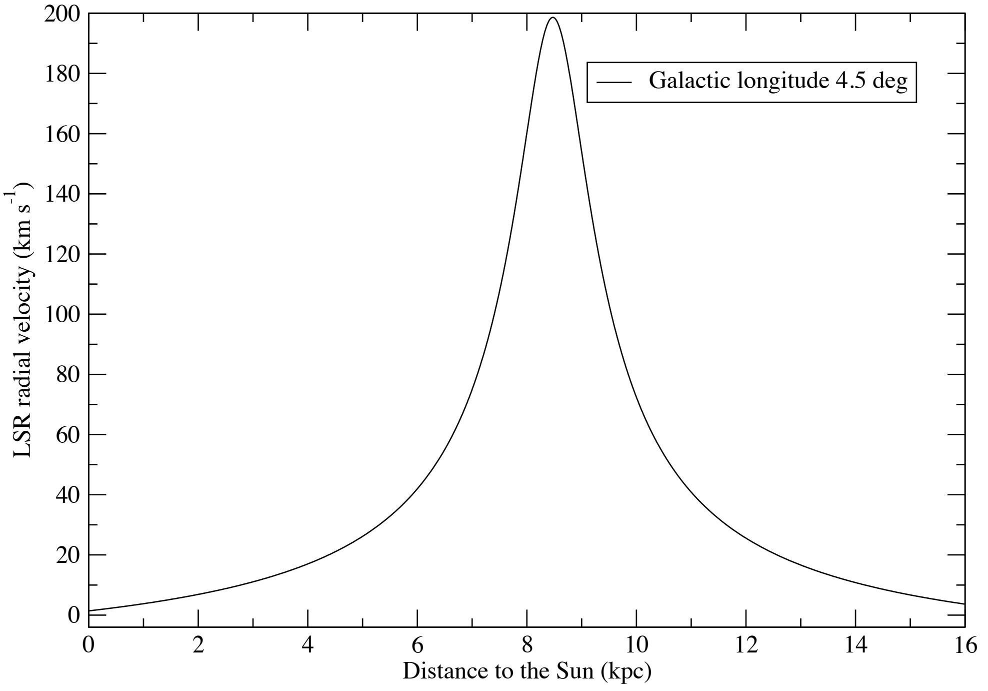

Supplementary Figure 2. Radial velocity along the line of sight as a function of distance. This plot has been computed using a standard Milky Way rotation curve[1] particularized to the Galactic longitude of GRS 1758-258. The emission peak of the CO cloud to the north of the microquasar is very close to the local standard of rest (LSR) maximum velocity of approximately 200 km s^{-1}, which corresponds to a distance of 8.5 kpc.

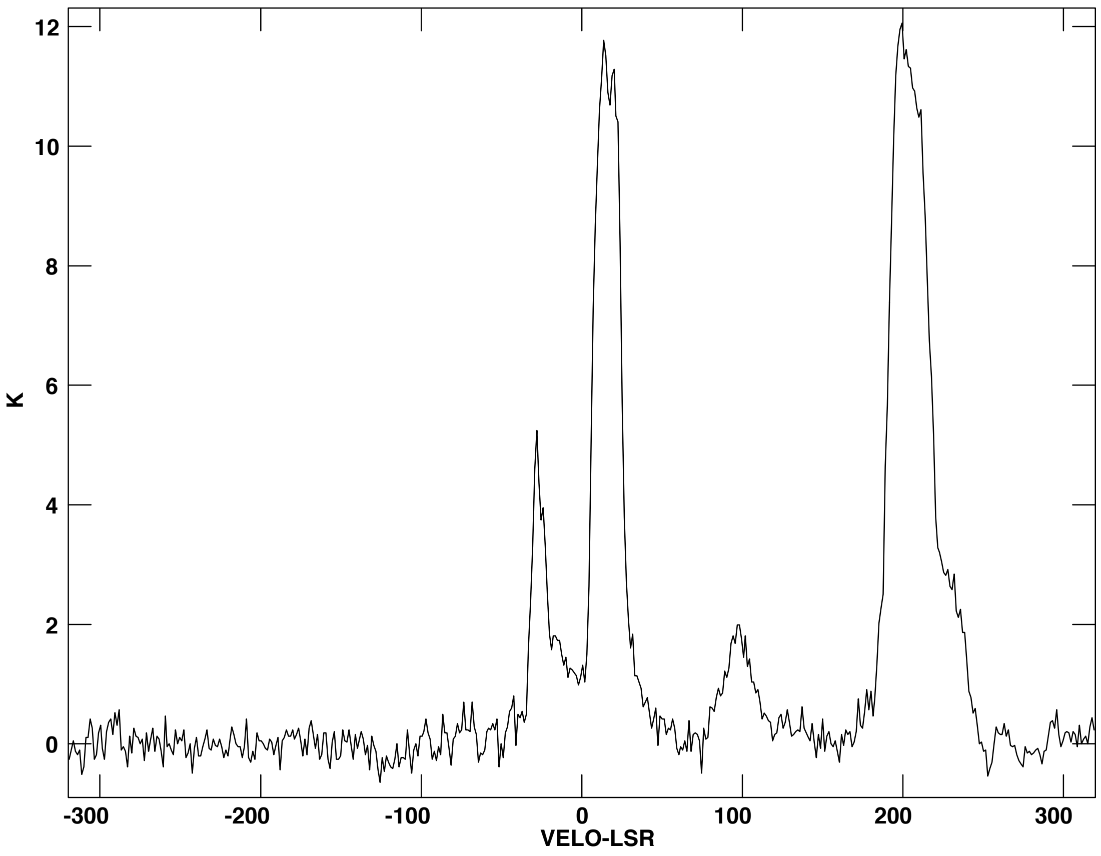

Supplementary Figure 3. Spectrum of CO emission. This radio spectrum was created from a data cube of a single dish CO survey[2] of the Galactic plane. It is centred at the emission peak of the CO cloud, which is north of GRS 1758-258. The horizontal axis represents the LSR velocity in km s^{-1}, and the vertical axis provides the antenna temperature in K. Several cloud components are evident at velocities of approximately -28, +14-20, +98 and +200-210 km s^{-1}, with the last component being the one that we associate with the microquasar.

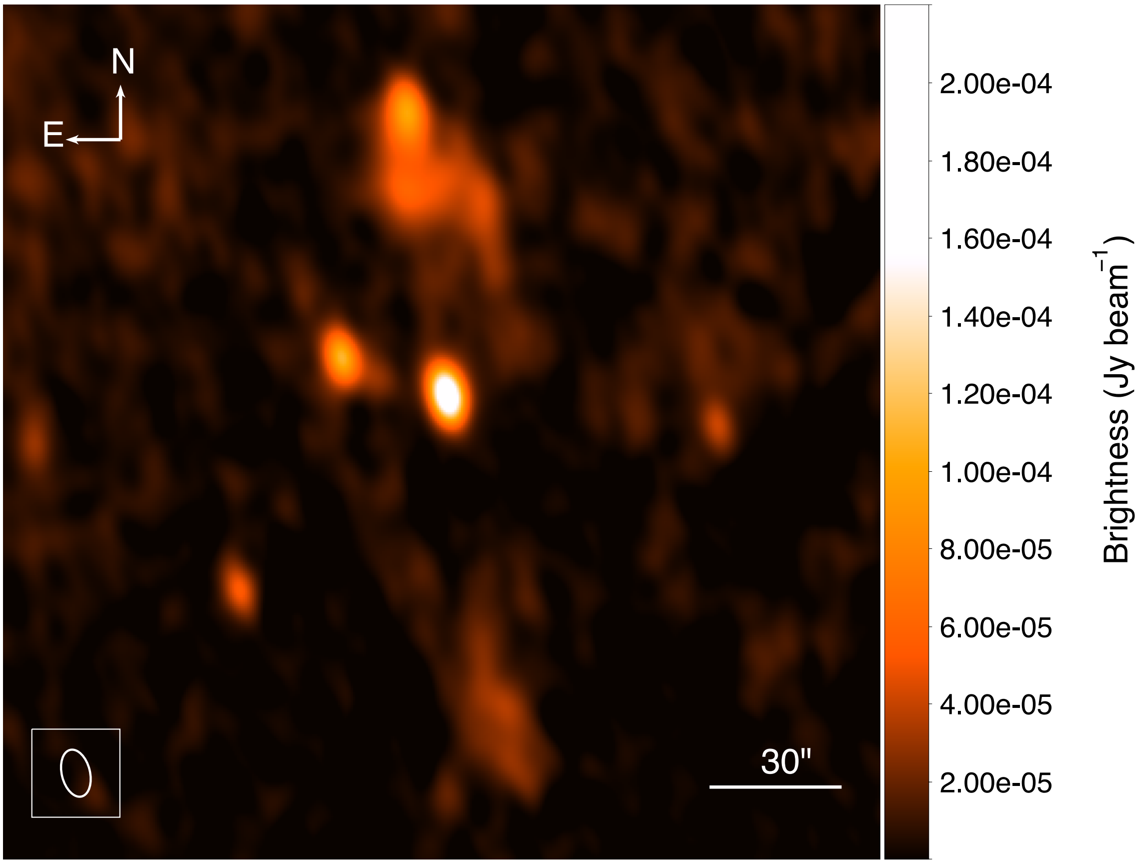

Supplementary Figure 4. 2016 radio appearance of the microquasar GRS 1758-258. This radio map at the 6 cm wavelength is the single-epoch map of 2016 obtained with the VLA in C configuration (see Methods section and Table 1). The Z-shaped morphology with natural weight is also visible in this single-epoch image, although not as clearly as in Figure 1. This is simply because short baseline visibilities from archival VLA data in its more compact D configuration have not been used here. The 30 arc-second horizontal bar indicates the angular scale. The right colour bar provides the brightness scale in units of Jy beam^{-1}. The synthesized beam (8.95 × 3.85 arc-second^2, with a position angle of 16°) is displayed by the ellipse in the bottom-left corner.

Supplementary Table 1. Central core and northern hot spot positions

Feature	Date	Right Ascension (J2000.0)	Declination (J2000.0)
Central core	1997	$18^h\ 01^m\ 12.39^s \pm 0.02^s$	-25° 44’ 36.5” ± 0.6”
	2008	$18^h\ 01^m\ 12.40^s \pm 0.02^s$	-25° 44’ 36.1” ± 0.4”
	2016	$18^h\ 01^m\ 12.391^s \pm 0.004^s$	-25° 44’ 36.24” ± 0.07”
Northern hot spot	1997	$18^h\ 01^m\ 13.00^s \pm 0.03^s$	-25° 43’ 33.5” ± 0.6”
	2008	$18^h\ 01^m\ 13.13^s \pm 0.03^s$	-25° 43’ 33.9” ± 0.5”
	2016	$18^h\ 01^m\ 13.08^s \pm 0.02^s$	-25° 43’ 31.3” ± 0.3”

Supplementary References

1. Verschuur, G. L., Kellermann, K. I., Galactic and Extra-Galactic Radio Astronomy, Springer-Verlag, p. 82 (1974)

2. Dame, T. M., Hartmann, D., Thaddeus, P., The Milky Way in Molecular Clouds: A New Complete CO Survey, *Astrophys. J.* **547**(2), 792-813 (2001)
